# The Impact of Chlorogenic Acid Liposomes Dip-Coating on the Physicochemical Quality and Microbial Diversity of Low-Salt Cured Fish During Refrigerated Storage

**DOI:** 10.3390/foods15020345

**Published:** 2026-01-17

**Authors:** Zixin Li, Yin Wang, Yong Jiang, Lili Chen, Meilan Yuan, Li Zhao, Chunqing Bai

**Affiliations:** National R&D Branch Center for Freshwater Fish Processing, College of Life Science, Jiangxi Science and Technology Normal University, Nanchang 330013, China; lzx146138@163.com (Z.L.); wangyin19970313@163.com (Y.W.); 1020130968@jxstnu.edu.cn (Y.J.); 1020140971@jxstnu.edu.cn (L.C.); 1020100975@jxstnu.edu.cn (M.Y.); 1020100962@jxstnu.edu.cn (L.Z.)

**Keywords:** lipid oxidation, microbial inhibition, refrigerated storage, chlorogenic acid, liposomes

## Abstract

Low-salt cured fish is prone to deterioration due to lipid oxidation and microbial proliferation during refrigeration. Chlorogenic acid (CGA), with excellent antioxidant and antimicrobial activity, is a promising candidate for the preservation of cured fish. However, its instability in the presence of environmental factors significantly confines its direct application. In this research, CGA was encapsulated in liposomes and utilized as a dip-coating for cured fish. The effects of varying concentrations of CGA-loaded liposomes (L-CGA) coating on the physicochemical quality and microbial diversity of cured fish were rigorously compared to those treated with CGA solutions, blank liposomes, and distilled water throughout 32 days’ storage at 4 °C. The results showed that L-CGA exhibited a higher lipid oxidation-inhibiting capacity (generation of hydroperoxides and their secondary oxidation products) than the corresponding free CGA at fixed concentrations. Furthermore, the liposomal formulation showed significantly enhanced inhibitory activity against dominant spoilage-associated bacterial genera (e.g., *Staphylococcus*, *Macrococcus*, and *Rothia*), with the L-CGA loaded at 800 mg/L of CGA showing optimal effectiveness. This enhanced preservation effect can be attributed to the protective and controlled release properties of the liposomes, which facilitate improved preservation outcomes for CGA. These findings demonstrate that L-CGA could be used as a promising preservative for low-salt cured fish or some similar products.

## 1. Introduction

Cured fish is a traditional aquatic delicacy appreciated worldwide for its distinctive flavor and firm texture [1]. Salting is an essential process in developing the unique characteristics of cured fish. Historically, a substantial amount of salt was required to create a high osmotic pressure environment for effectively inhibiting microbial growth and lipid oxidation during storage [2], thereby resulting in a high salt content (20–30%) in cured fish. However, excessive dietary salt intake is associated with significant health risks, including hypertension, coronary heart disease, etc. [3,4]. Consequently, the development of low-salt cured fish products has become a major focus within the food industry. To date, although various methods have been devised to produce low-salt products with traditional characteristics, reducing salt content introduces a significant challenge: it diminishes the inherent antimicrobial and antioxidant protection provided by high salt. As a result, low-salt cured fish becomes more susceptible to rapid microbial spoilage and accelerated lipid oxidation during storage [5,6]. These processes collectively lead to quality deterioration, off-odor development, and markedly reduced shelf-life. Therefore, finding effective preservation strategies to maintain the quality and extend the storage stability of low-salt cured fish is urgently needed.

The application of antioxidants and bacteriostatic agents in coating cured fish can mitigate these challenges to some extent [7]. Various synthetic antioxidants, such as butylated hydroxyanisole (BHA), butylated hydroxytoluene (BHT), and tertiary butylhydroquinone (TBHQ), along with bacteriostatic agents like sodium sorbate and sodium dehydroacetate, are extensively utilized in the food industry due to their cost-effectiveness, large-scale production, and efficacy [8,9,10]. However, researchers found that the aforementioned antioxidants and bactericides posed certain toxic side effects, with prolonged consumption potentially elevating the risks of carcinogenesis, teratogenesis, and mutagenesis [9]. Consequently, the development of natural coating preservatives with robust antioxidant and bacteriostatic properties has attracted increasing attention in recent years.

Polyphenols represent a major class of plant secondary metabolites distinguished by multiple phenolic hydroxyl groups. This broad category encompasses phenolic acids, flavonoids, stilbenes, curcuminoids, lignans, and tannins, which are ubiquitously present in fruits, vegetables, legumes, cereals, and tea [11]. Owing to their potent antioxidant and antimicrobial activities, coupled with recognized health benefits, including anticancer, antitumor, and cardiovascular protective effects, polyphenols have attracted substantial interest from both the scientific community and consumers [9]. In recent years, their application in preserving and extending the shelf-life of meat products has become a research hotspot. For example, Cao et al. [12] demonstrated that chlorogenic acid effectively slowed lipid and protein oxidation in grass carp. Similarly, Yang et al. [13] reported that catechins significantly inhibited lipid oxidation and maintained quality in pork.

However, a critical limitation hindering the direct application of polyphenols is their inherent sensitivity to environmental factors such as light, temperature, and oxygen, leading to rapid degradation and a consequent loss of sustained efficacy [14]. To address this challenge, encapsulation within protective delivery systems, such as liposomes, microspheres, or emulsions, has been proposed to enhance stability and enable controlled release. Among them, liposomes have emerged as particularly promising carriers due to their biocompatibility, protective capacity, and ability to modulate the release of bioactive compounds [15]. While liposomes have been widely studied for encapsulating various actives (e.g., polyphenols, vitamins), research remains predominantly focused on their preparation, characterization, and release kinetics. Their practical application as carriers for natural preservatives in complex food matrices, especially in traditional, semi-dry products, is still largely underexplored.

Chlorogenic acid (CGA), a key polyphenol abundant in plants such as honeysuckle and coffee, is renowned for its strong antioxidant and antimicrobial properties, alongside anti-inflammatory and cardiometabolic health benefits [16]. Its colorless and odorless nature makes it particularly suitable for food applications [17]. Although CGA has shown efficacy in preserving various foods, including fruits, vegetables, tilapia, and carp [16], its use in traditional low-salt cured fish remains virtually unreported. Moreover, the unique physicochemical and microbial ecosystem of cured fish differs markedly from that of fresh aquatic foods after the salting and drying process, thereby necessitating tailored preservation approaches. Crucially, the potential of liposome-encapsulated CGA (L-CGA) to overcome stability limitations and provide sustained protection in such a challenging matrix has not been investigated.

To bridge this knowledge gap, the efficacy of L-CGA dip-coating on the physicochemical quality and microbial dynamics of low-salt cured fish during 32 days of storage at 4 °C was systematically evaluated in this work. By integrating physicochemical analysis, conventional microbiological assays with 16S rRNA high-throughput sequencing, a comprehensive analysis of how this innovative delivery system modulates spoilage microbiota and product stability would be provided. This work would offer fundamental insights into the application of polyphenol-loaded liposomal preservation strategy in cured fish or similar aquatic products.

## 2. Materials and Methods

### 2.1. Materials

Fresh grass carp (*Ctenopharyngodon idella*, from which post-mortem filets were obtained for the study) and food-grade salt were purchased from a local supermarket (Nanchang, China). CGA (≥98% purity), egg yolk lecithin (phosphatidylcholine content ≥ 90%), and cholesterol were obtained from Shanghai Aladdin Biochemical Technology Co., Ltd. (Shanghai, China). Analytical-grade chemicals, including thiobarbituric acid (TBA), trichloroacetic acid (TCA), and sodium chloride, were supplied by Shanghai Sangon Biotech Co., Ltd. (Shanghai, China). All other chemicals were of reagent grade.

### 2.2. Preparation of Cured Fish Samples

Fresh grass carp (without scales and internal organs) was cut into uniformly sized pieces (each weighing 20 ± 0.50 g) and immersed in a 6% (*w*/*v*) saline solution. After 6 h, the fish pieces were drained and subjected to a two-step drying (oven dried at 60 °C for 12 h and then dried for an additional 48 h at 8 °C and 45% humidity) [18,19]. The cured fish was then randomly divided into ten groups. Each group was fully immersed in its respective coating solution. Four groups immersed in CGA aqueous solutions at concentrations of 400, 600, 800, and 1000 mg/L were named as 400, 600, 800, and 1000, respectively. Another four groups immersed in L-CGA at the same CGA concentrations were designed as L-400, L-600, L-800, and L-1000, respectively. The remaining two groups were treated with blank liposome (BL) and distilled water (Water, control), respectively. After immersion for 5 min, the samples were withdrawn, allowed to drain briefly, and then air-dried under ventilation at 25 °C for 30 min to form a stable coating layer on cured fish pieces. All samples were subsequently stored at 4 °C in the dark for further analysis. It is important to note that the concentration range of CGA employed in this study (400–1000 mg/L) was selected based on a comprehensive synthesis of existing literature and our preliminary experiments. Previous research has indicated that lower concentrations (below 500 mg/L) exhibited limited antioxidant effects, whereas higher concentrations (1000–3000 mg/L) demonstrated significant preservative efficacy when CGA was used directly [12]. Our preliminary experiments further revealed that encapsulating chlorogenic acid in liposomes (L-CGA) could effectively extend its duration of action while lowering its required effective dose. However, the preservation effect was found to be dose-dependent within a specific range (within 1000 mg/L), beyond which further increases in concentration did not result in substantial additional benefits. Consequently, a concentration range of 400–1000 mg/L was selected to effectively assess the advantages of encapsulation while maintaining experimental relevance and efficiency.

### 2.3. Preparation of L-CGA

L-CGA was prepared using the ethanol injection method [20]. Briefly, egg yolk lecithin, cholesterol, and CGA (100:20:12, *w*/*w*) were dissolved in ethanol. After complete dissolution, the solution was gradually injected into phosphate-buffered saline (PBS, 0.02 M, pH 7.2) under continuous stirring at room temperature for 30 min. Ethanol was then removed by rotary evaporation (N-1100, EYELA, Tokyo, Japan) under vacuum at 40 °C. Finally, the resulting suspension was brought back to the original volume with distilled water and ultrasonicated for 20 min to obtain a uniform L-CGA.

### 2.4. pH Measurement

At predetermined storage time intervals (0, 8, 16, 24, 32 days), the cured fish pieces were taken out and crushed. 2 g of the crushed fish was mixed with 20 mL of distilled water and homogenized for 1 min. The pH of the resulting supernatant was measured using an electronic pH meter (PHSJ-5, INESA, Shanghai, China).

### 2.5. Peroxide Value Measurement

The peroxide value (POV) was determined following the method outlined by Vareltzis et al. [21] with modifications. Specifically, 2 g of cured fish meat was combined with 15 mL of a methanol-chloroform solution (1:2, *v*/*v*) and homogenized on a blender (Vortex-Genie, Naze Co., Ltd., Shanghai, China) at 13,500 rpm for 30s. Subsequently, 3 mL of a 0.5% NaCl solution was added, and the mixture was centrifuged at 3000 rpm for 10 min. 5 mL of the lower-phase liquid was then taken out, combined with an equal volume of methanol-chloroform mixture (1:2, *v*/*v*), and mixed thoroughly. Consequently, 25 μL of ferric chloride solution (3.5 g/L) and ammonium thiocyanate solution were incorporated individually into the mixture. After allowing the samples to stand at room temperature for 5 min, their absorbance was measured at a wavelength of 500 nm. The POV was subsequently calculated using Equation (1):

(1) $$\mathrm{POV}\;(\mathrm{meq}/\mathrm{kg})\;=\frac{C}{M\times 55.84}$$

where C represents the concentration of iron in the sample, calculated based on the standard curve of the iron solution. M denotes the sample mass, and 55.84 is the atomic mass of iron.

### 2.6. Thiobarbituric Acid Reactive Substances Analysis

The thiobarbituric acid reactive substances (TBARS) analysis was determined according to the method of De Leon & Borges [22]. In brief, 5 g of crushed fish was mixed with 10 mL of 7.5% trichloroacetic acid solution (containing 0.1% EDTA) and homogenized at 13,500 rpm for 2 min. The mixture was subsequently centrifuged, and 5 mL of the supernatant was combined with 5 mL of TBA solution (0.02 mol/L), heated at 90 °C for 40 min, and then allowed to cool to ambient temperature. Subsequently, the sample was subjected to post-centrifugation at 5000 rpm for 10 min. A volume of 5 mL of the supernatant was combined with an equal volume of chloroform, vortexed for 60 s, and allowed to stand for 5 min. The absorbance of the upper phase was then measured at 532 nm (A_532_) and 600 nm (A_600_), respectively. TBARS values were calculated using Equation (2).

(2) $$\mathrm{TBARS}\;(\mathrm{mg}\;\mathrm{MDA}\;\mathrm{eq}/\mathrm{kg})=\frac{\left(A_{532}-A_{600}\right)\;\times \;72.6\;\times \;100}{155\;\times \;10}$$

where 155 is the molar absorption coefficient of malondialdehyde (MDA), and 72.6 denotes the relative molecular weight of MDA.

### 2.7. The Traditional Microbial Culture

At the end of the 32-day storage period, 10 g of cured fish samples, ground using a sterile blender, were accurately weighed and combined with 90 mL of sterile normal saline. After patting and homogenization, the samples were serially diluted using sterile saline. The diluted samples were then smeared onto the surface of agar plates. The dishes were incubated at 30 °C for a duration of 48 h [23], and the growth of the bacterial population was observed every 12 h intervals. Based on variations in growth time, coloration, and the morphological characteristics of the bacterial colonies, continuous isolation and purification were performed until a homogeneous bacterial population was achieved. The isolated colonies were then transferred into sterile centrifuge tubes for microbial diversity identification.

### 2.8. 16S rRNA High-Throughput Sequencing

At the end of the 32-day storage period, samples were cut into small pieces using sterile scissors, and an appropriate amount was placed in sterile tubes. DNA of samples was extracted in a tissue lyser, and the DNA served as a template for PCR amplification. After purification via agarose gel electrophoresis, the PCR products were quantitatively measured using Quant-iT™ dsDNA HS Assay Kit (Thermo Fisher Scientific, Shanghai, China). The sequencing of the samples was performed on the Illumina HiSeq 2500 platform provided by Sangon Biotech (Shanghai, China) Co., Ltd. (Shanghai, China). The Tags sequences were subjected to a clustering operation using the UCLUST algorithm within QIIME (Version 1.7.0). When the similarity was ≥97%, the number of Operational Taxonomic Units (OTUs) was obtained. An Alpha diversity analysis was then carried out on the samples. The commonly used α-biodiversity indices, such as the Shannon index, Chao1 index (species richness index), Simpson index, coverage, and ACE, were calculated using QIIME (Version 1.7.0). Additionally, relative abundance bar charts of microorganisms at the family and genus level, a relative abundance heat map at the phylum and genus level, and a diagram of taxonomic system composition of microflora were completed on the Biomarker bio-cloud platform.

### 2.9. Statistical Analysis

All experiments were conducted with at least three independent biological replicates, and the data are presented as the mean ± standard deviation. Statistical analysis was performed using one-way analysis of variance (ANOVA) on SPSS software (Version 17.0). Where ANOVA indicated significant differences, Duncan’s multiple range test was applied for post hoc comparisons. A *p*-value of less than 0.05 was considered statistically significant.

## 3. Results and Discussions

### 3.1. The pH Measurement

**Figure 1:**
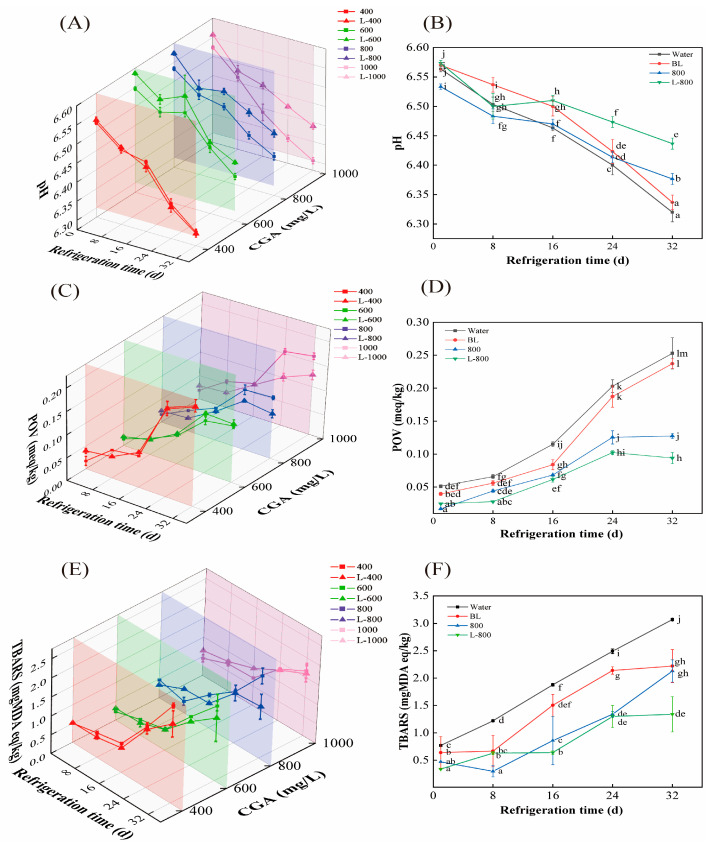
The effects of dip-coating on the pH (**A**,**B**), POV (**C**,**D**), and TBARS (**E**,**F**) of cured fish during 32-day refrigerated storage. Different treatment groups with different letters were significantly different (*p* < 0.05).

pH is a vital index for assessing the physicochemical qualities of aquatic products [24]. Lower pH means more acidic components are generated in samples. Figure 1A shows the pH changes in different groups over 32 days’ storage. Clearly, the initial pH values of the Water and BL groups were 6.56 and 6.57, respectively. When coated with 400–1000 mg/L CGA solution, the values declined to 6.52, 6.55, 6.53, and 6.52, respectively. Interestingly, minimal pH decline was observed in the groups treated with L-CGA. According to literature, CGA is an acidic compound that may release protons (H^+^) upon coating on cured fish, thereby potentially lowering the pH value [25]. However, due to the effective encapsulation of CGA within liposomes, its ability to release protons was significantly reduced, resulting in a negligible impact on the pH values. Over the 32-day storage period, the pH of all groups progressively decreased, indicating the generation of acidic components in all samples. This is consistent with the changes observed in beef patties, as reported by Tarasevičienė et al. [26]. Specifically, the pH of the Water and BL groups decreased to 6.32 and 6.34 at the end of storage, respectively. Although similar trends were observed for the CGA solutions and L-CGA-treated groups, the values exhibited dependency on CGA concentration and its encapsulation type. Furthermore, the pH of both CGA and liposome-treated samples was higher compared to the controls (BL group) at fixed time points, with the liposome-treated samples showing a more pronounced effect. The pH of samples treated with CGA solutions (400–1000 mg/L CGA) decreased to 6.31, 6.39, 6.38, and 6.30, respectively, after 32 days’ storage. Obviously, the reduction in pH magnitude decreased with increasing concentrations of CGA in the range of 400–800 mg/L; however, a further increase in reduction was observed once the concentration exceeded 800 mg/L. Comparable outcomes were observed in the L-CGA-treated groups (L-400 to L-1000), with the L-600 and L-800 groups demonstrating the smallest decline in pH values.

During storage, the glycolytic process and lipid oxidation and hydrolysis may co-occur in cured fish. On one hand, the progress of the glycolytic process may gradually produce lactic acid [27]. On the other hand, the oxidation and hydrolysis of lipid may generate acidic components, such as free fatty acids or carboxylic acid oxidation compounds [28]. Additionally, the metabolic activities of microorganisms present in the fish may also contribute to the continuous generation of acidic substances. These factors collectively cause a decrease in the pH value of cured fish during storage.

As a prominent type of polyphenol, CGA, with five active hydroxyl groups and one carboxyl group within its molecular structure, exhibits remarkable antioxidant and antibacterial properties [17]. The observed lower changed pH during storage for the CGA and L-CGA treated groups, as compared to the controls, can likely be attributed to the antioxidant and antibacterial properties of CGA. These properties may play a significant role in slowing down lipid oxidation and inhibiting microbial growth, thereby resulting in the generation of fewer acidic compounds [29]. Higher concentrations of CGA (400–800 mg/L) may enhance these antioxidant and antibacterial activities, leading to a smaller decline in pH. However, when the antioxidant content surpasses a certain threshold, further increases in concentration yield minimal improvements in antioxidant capacity. Whereas, a portion of CGA may undergo oxidation or degradation, thereby resulting in not ideal preserve effect. Some oxidation products of CGA, such as quinones, may even accelerate the formation of free radicals, hastening lipid oxidation and increasing the production of acidic substances, which may further result in a more pronounced decrease in pH values [29]. Consequently, both the CGA solution groups and L-CGA groups exhibited an optimal concentration that showed better quality preservation. As depicted in Figure 1B, the L-800 and 800 treated groups maintained a higher pH at the end of the storage period compared to other groups within the same types of coating systems. In addition, it may be the protective effect and sustained-release properties of liposomes for CGA that ensured the high activity of CGA, thereby improving the quality of L-CGA-treated groups compared to the corresponding CGA solution groups (particularly for L-800).

### 3.2. Peroxide Value Analysis

Lipid oxidation is a primary cause of quality deterioration in meat products. Quantitative determination of the degree of lipid oxidation is crucial for evaluating the quality of cured fish [30].

As illustrated in Figure 1C, the POV increased in all samples throughout storage, indicating continuous generation of hydroperoxides [31]. Specifically, the POV exhibited a gradual increase during the initial 16 days, followed by a significant rise. This phenomenon could be attributed to unsaturated fatty acids in cured fish that would undergo auto-oxidation, which is a free radical chain reaction, and could be divided into the initial period, propagation, and termination [30]. After 16 days, lipid oxidation likely entered the propagation phase, in which each radical can initiate multiple chain reactions via hydrogen abstraction [32], leading to accelerated hydroperoxide accumulation and a sharp increase in POV.

After 32 days of storage, the L-CGA groups (except for the L-400 group) showed a significantly smaller increase (*p* < 0.05) in POV than the corresponding CGA groups. For instance, the POV of the 800 group increased from 0.017 to 0.127 meq/kg, while that of the L-800 group increased from 0.025 to 0.094 meq/kg. These results confirm that liposomal encapsulation effectively enhances the antioxidant efficacy of CGA.

Changes in POV across treatment groups are summarized in Figure 1D. After 32 days, the POV of the BL and Water groups increased from 0.040 to 0.237 meq/kg and from 0.051 to 0.253 meq/kg, respectively. The relatively lower POV in the BL group may be attributed to the inherent antioxidant activity of yolk phospholipids [33]. Although the POV trends in the 800 and L-800 groups were similar to the BL group, their POVs were significantly lower at each time point. Moreover, the L-800 group exhibited the lowest final POV (0.094 meq/kg) among all groups, consistent with the pH results, underscoring its strong potential in suppressing lipid oxidation in cured fish.

### 3.3. Thiobarbituric Acid Reactive Substances Analysis

As previously discussed, the hydroperoxides formed during the oxidation of unsaturated fatty acids undergo further oxidation and decomposition into secondary products, such as hydrocarbons, aldehydes, and other low molecular weight compounds, which can be quantified using the TBARS assay [34]. It is widely accepted that these secondary lipid oxidation products are primarily responsible for the development of off-flavors in meat [35]. In this section, we monitored the levels of secondary oxidation products in cured fish subjected to various treatments.

As illustrated in Figure 1E, the TBARS values for all groups of cured fish increased throughout the storage period, indicating lipid oxidation progressed as the time prolonged. Notably, the TBARS values for samples treated with Water and BL were significantly higher than those treated with CGA solutions and L-CGA at any given storage time, suggesting that both CGA and L-CGA effectively delay lipid oxidation in cured fish. Furthermore, the increase in TBARS values for samples treated with CGA solutions and L-CGA was slower during the initial period of storage. This observation can be attributed to two primary factors. Firstly, the majority of fatty acids in the cured fish remain unoxidized, resulting in a relatively low generation of secondary oxidation products during this stage. Secondly, the antibacterial and antioxidant properties of CGA likely inhibited the formation of these secondary oxidation products. However, after 8 days of storage, there is a rapid increase in TBARS levels, followed by a continued gradual rise. The rapid increase in TBARS indicates the generation of more secondary lipid oxidation products. This phenomenon may be attributed to elevated oxygen levels due to dehydration during microbial reproduction, which may facilitate the rapid accumulation of lipid oxidation products [30]. The subsequently slower increased TBARS values in the later stages of storage may be explained by the proliferation of lactic acid bacteria (LAB), which may produce significant amounts of catalase, thereby slowing down lipid oxidation [36]. Moreover, it has been reported that secondary oxidation products, such as aldehydes and ketones, are prone to volatilization or degradation during storage, contributing to a decrease in TBARS values [30]. Additionally, certain nucleophilic aldehydes, such as MDA, may interact with proteins to form Schiff base compounds, potentially slowing the accumulation of aldehyde [37]. It is important to note that nearly no significant difference was observed between the CGA and L-CGA groups at the initial stage of storage. However, a substantial difference emerged by the end of the storage period. CGA, known for its excellent antioxidant capacity, may effectively inhibit lipid oxidation in the early stages. Nonetheless, its antioxidant activity may diminish over time, likely due to self-degradation. In contrast, L-CGA, benefiting from the protective and controlled release properties of the liposomal bilayer, demonstrated prolonged antioxidant activity. Among the various concentrations of L-CGA, the L-800-treated samples exhibited the lowest TBARS values (1.34 mg MDA eq/kg), consistent with the POV and pH results.

### 3.4. The Traditional Microbial Culture

**Figure 2:**
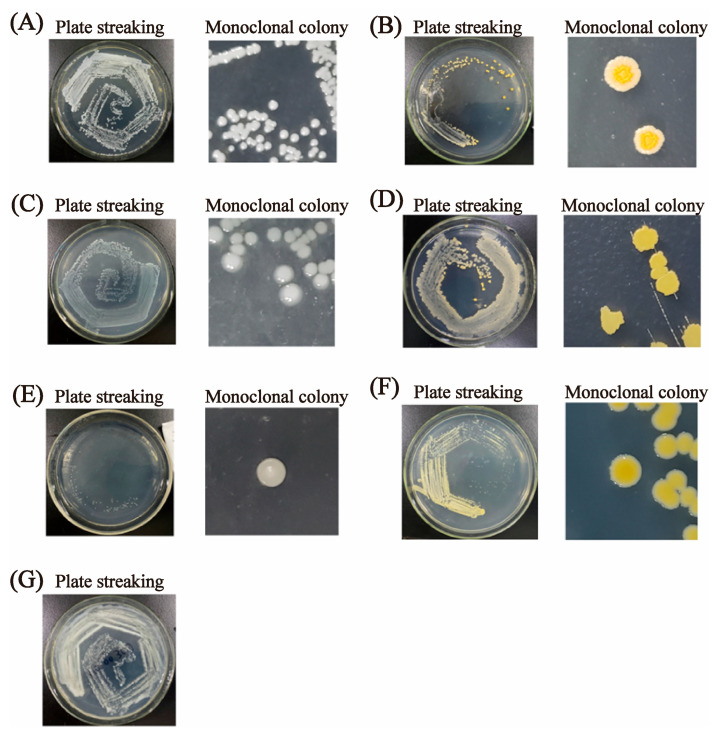
The morphology of seven dominant colonies isolated from cured fish. (**A**) *Blautia hydrotrophica*; (**B**) *Staphylococcus succinus* DZXOTU3; (**C**) *Enterococcus faecalis* GI60; (**D**) *Staphylococcus succinic* strain JM40; (**E**) Unidentified; (**F**) *Staphylococcus saprophyticus;* (**G**) *Enterobacter hormaechei*.

Although the composition of bacterial colonies varied slightly among the ten cured fish samples, seven dominant strains were isolated. Their growth status after 12 h is shown in Figure 2 (note: strain G grew too rapidly, resulting in overgrown and adherent colonies that prevented clear observation of individual morphologies, and thus is not displayed). The identification results and morphological characteristics of each strain are summarized in Table 1.

Staphylococcus was identified as the predominant genus in the samples, which is consistent with previous studies on bacterial diversity in cured products from Longxi, China [38]. Although lactic acid bacteria (LAB) play a critical role in flavor development in cured foods [39], they were not detected in this study. This may be attributed to the facultative anaerobic nature of LAB [40]. During the initial stages of pickling, the high moisture content in meat and low oxygen (consumed by numerous microorganisms) conditions may create a favorable environment for LAB growth. However, as the pickling process progressed, reduced moisture content (meat became desiccated), pulsed increased *Staphylococcus* populations may greatly inhibit the proliferation of LAB. Additionally, the cultivation medium may have influenced the detection of LAB. In this work, to isolate and identify the dominant bacteria in cured fish, the microbes were cultivated on plate count agar medium at 30 °C. This environment may not be optimal for LAB, which ideally requires MRS agar medium and an incubation temperature of 37 °C [40].

It is worth noting that among the seven isolated strains, three strains may contribute positively to the development of cured fish flavor. Firstly, *Enterococcus faecalis*, with the capability to acidify and proteolytically hydrolyze proteins, has been widely applied to produce pickled or fermented dairy products [41]. Its association with meat flavor often stems from its ability to produce lactic acid and other acidic compounds, contributing to the sour notes in fermented meat products. Secondly, *Staphylococcus succinus*, recognized as a safe and non-toxic strain with robust growth potential, could enhance the expression of genes related to glycolysis and amino acid metabolism, thereby promoting the synthesis of free amino acids associated with sweet and umami tastes, and improving the flavor and quality of fermented food [42]. Thirdly, *Staphylococcus saprophyticus* has been documented to enhance the decomposition of fats and proteins in pickled foods. Yang utilized *S. saprophyticus* as a fermentation agent in Chinese bacon [43], which significantly increased the product’s redness and water activity. However, excessive lipid oxidation can produce various small-molecule metabolites that not only diminish food quality but also pose food safety risks due to the accumulation of aldehydes and other substances.

Furthermore, certain bacteria have been identified that inhibit the growth of spoilage bacteria in cured fish through the production of antimicrobial substances. For instance, bacteriocins metabolized by *E. faecalis* have been shown to inhibit the growth of *Pseudomonas*, *Salmonella*, *Shigella*, and *Listeria*. This inhibitory effect is primarily attributed to the ability of the bacteriocins to bind to surface receptors on these bacteria, activate their ion channels, and cause significant leakage of cellular ions. This disruption impairs essential life functions and hinders nutrient transport, ultimately leading to bacterial cell death [44]. The increase in *E. faecalis* populations observed in the control group may be of concern, given that some strains of *E. faecalis* are recognized as opportunistic pathogens in clinical settings [45]. However, it is important to note that this study did not directly assess the pathogenicity or health effects of the specific strains present in the cured fish. The implications of this increase for human health, therefore, require further investigation.

During the experimental process, it was observed that the dominant bacterial communities varied slightly among the 10 sample groups. For instance, *Blautia hydrotrophica*—a critically important anaerobic bacterium in the gut [46], primarily responsible for fermenting dietary fiber and producing short-chain fatty acids (e.g., acetate) essential for maintaining gut health [45] was detected in the Water-treated group. Conversely, in the BL group, *Enterobacter hormaechei* was present at relatively higher levels, which may be attributed to the lack of suppression by CGA and the low-salt environment that failed to inhibit its proliferation.

### 3.5. 16S rRNA High-Throughput Sequencing

#### 3.5.1. Alpha Diversity Analysis

The microbial communities in cured fish were significantly complex. In this section, the richness and evenness of microbial communities within different groups were analyzed by using alpha diversity in terms of Chao1, Ace, Shannon, Simpson, and coverage. Among these, Ace and Chao1 indices were used to evaluate species abundance, with higher values indicating a greater number of species. The Simpson and Shannon indices were utilized to assess species diversity, where a higher Shannon index and a lower Simpson index indicate greater species diversity within the sample [47]. The Coverage value represents the coverage rate, with values approaching 1 indicating a higher likelihood of detecting species in the sample [48].

As illustrated in Table 2, the coverage for each group was higher than 0.99, suggesting that the microbial sequences were effectively captured and that the sequencing depth met the requirements for diversity analysis. Furthermore, the OTU numbers derived from the Chao and Ace algorithms exhibited substantial consistency. In general, the OTU numbers were elevated in cured fish samples treated with 600 and 800 mg/L CGA solutions, as well as in samples treated with L-400. Conversely, the OTU counts were comparatively lower both in the 1000 group and the BL group. Additionally, the microbial diversity assessments measured by both the Shannon and Simpson indices were largely consistent. Notably, microbial diversity was significantly elevated in cured fish treated with 600 and 800 solutions, as well as L-400, which corresponded with the higher OTU counts in these samples. Conversely, the lowest microbial diversity was recorded in cured fish coated with L-800. It is crucial to acknowledge that the microbial diversity is highly complex in cured fish, with phylogenetic relationships among different microorganisms often exhibiting significant variability. Therefore, microbial communities analyzed based on setting microbial species as independent variables are insufficient. Further investigation is warranted to explore the scientific impact of various treatments on microbial communities in cured fish, particularly regarding species abundance diversity across different cured fish groups.

#### 3.5.2. Differences in Species Abundance Distribution Among Different Groups of Cured Fish

**Figure 3:**
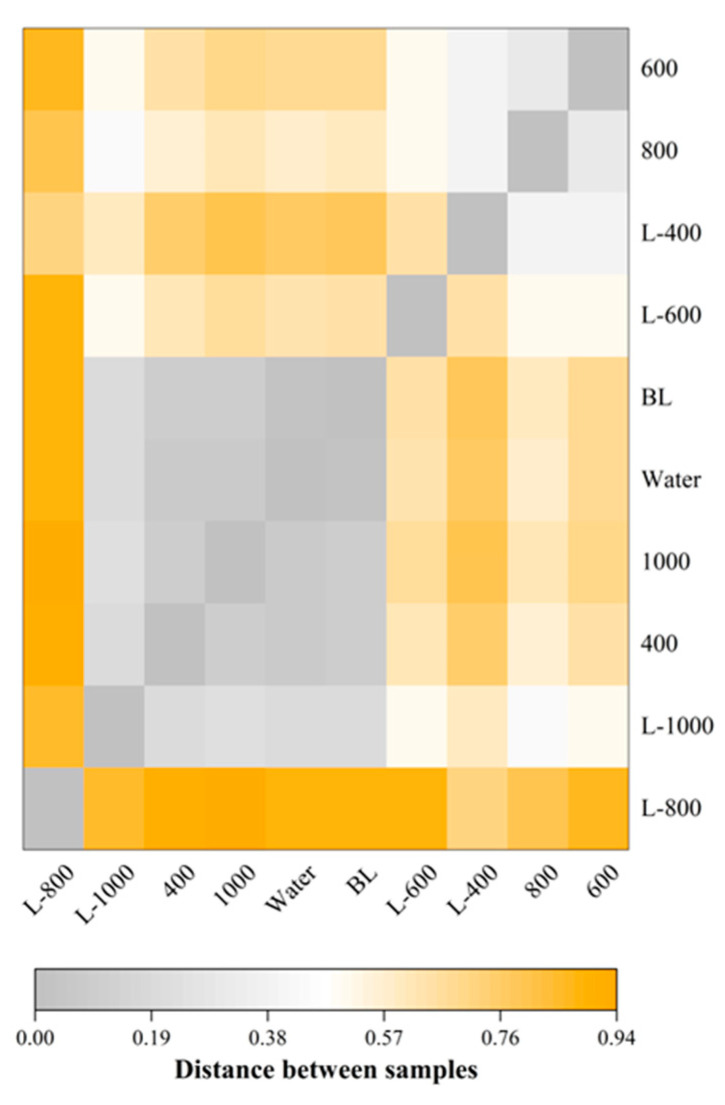
Differences in species abundance distribution across ten groups of cured fish.

To evaluate the effect of different antioxidant treatments on the microbial composition of cured fish, we quantified intergroup differences in species abundance distribution using statistical distance metrics. As illustrated in Figure 3, the species abundance distribution of the BL group was similar to that of the Water group, suggesting that BL had negligible antimicrobial activity. Among the CGA-treated groups, the microbial profiles of the 400 and 1000 groups closely resembled each other and were similar to those of the BL and Water groups. In contrast, the 600 and 800 groups formed a separate cluster, with abundance distributions markedly different from the control groups. These results imply that 600 and 800 possessed stronger antimicrobial efficacy, whereas concentrations lower or higher than this range showed reduced effectiveness.

In the L-CGA-treated groups, the microbial composition of L-1000 was similar to that of 400 and 1000. The L-400 and L-600 groups, however, clustered closely with 600 and 800, respectively, indicating that high concentrations of liposome-encapsulated CGA may also lead to reduced antimicrobial performance. Furthermore, at the same CGA concentration, the beta-diversity distance between the CGA groups and the Water group was significantly smaller than that between the corresponding L-CGA groups and the Water group. This supports the view that liposomal encapsulation enhances the antibacterial activity of CGA, likely by improving its stability and providing protection against degradation. It is also noteworthy that the species distribution in the L-800 group was distinct from all other groups, which may be attributed to the strongest antibacterial activity of L-800, effectively inhibiting a broader spectrum of microorganisms.

#### 3.5.3. Differences in the Abundance Distribution of Dominant Microflora Among Different Groups of Cured Fish (At the Phylum Level)

**Figure 4:**
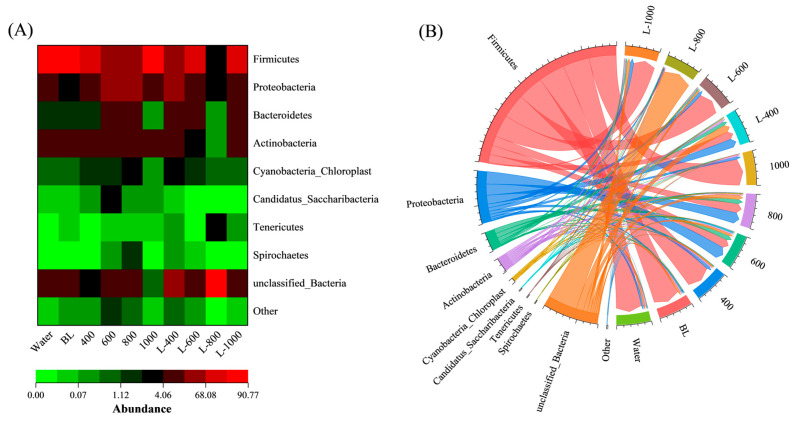
Phylum-level microbial community composition (**A**) and its correlation with the various treatment groups (**B**) in cured fish.

Figure 4A shows the relative abundance of dominant bacterial phyla across the ten cured fish groups in a heatmap, where rows represent phyla, columns represent samples, and color intensity indicates relative abundance (red: higher; green: lower). Firmicutes was the most abundant phylum in most groups, followed by Proteobacteria, Bacteroidetes, and Actinobacteria.

To further elucidate the proportional distributions of dominant microflora across different groups, a collinearity analysis was performed on the ten groups of cured fish. As depicted in Figure 4B, the right semicircle illustrates the bacterial community abundance in various cured fish samples, whereas the left semicircle depicts the distribution proportion of these bacterial communities across different cured fish groups. Obviously, the Water and BL groups exhibited similar dominant microbial profiles, with Firmicutes showing the highest proportions (81.56% and 82.64%), followed by Proteobacteria (6.17% and 4.05%), Bacteroidetes (1.57% and 1.76%), Actinobacteria (4.36% and 4.40%), Cyanobacteria_Chloroplast (0.92% and 0.72%), and Candidatus_Saccharibacteria (0.05%, 0.03%). This phenomenon further demonstrates that BL do not exert a significant inhibitory effect on the microbial communities presented in cured fish.

Firmicutes, constituting the predominant microbial phylum in traditional cured fish, can be classified into three classes: Clostridia, Bacilli, and Mollicutes [49]. Notably, the genera Enterococcus and Lactobacillus within the Bacilli class are heat-resistant strains, enabling their survival in cured fish. This characteristic may contribute to the relatively high abundance of the Firmicutes phylum.

In contrast, the groups treated with CGA solutions exhibited considerable reductions in Firmicutes abundance: 79.01% (400), 27.78% (600), 29.82% (800), and 84.07% (1000). These findings also suggest that CGA possesses an inhibitory effect on the proliferation of Firmicutes, with the 600 and 800 groups demonstrating a more pronounced inhibitory effect. Conversely, both low (400) and high (1000) concentrations of CGA exhibit relatively weak antibacterial effects on Firmicutes. This concentration-dependent effect aligns with the pH and lipid oxidation trends, suggesting that insufficient inhibition of Firmicutes (particularly spoilage-associated Bacillus [50]) at suboptimal CGA levels may accelerate proteolysis and lipolysis, leading to a more pronounced pH drop.

Moreover, L-CGA groups further reduced microbial abundance compared to CGA groups. The L-800 group showed the strongest suppression, with Firmicutes accounting for only 3.22%. Other dominant phyla were also markedly reduced: Proteobacteria (2.53%), Bacteroidetes (0.32%), Actinobacteria (0.37%), Cyanobacteria_Chloroplast (0.65%), and Tenericutes (2.12%). These results demonstrate that liposomal encapsulation enhances the antimicrobial efficacy of CGA, with L-800 being the most effective formulation.

#### 3.5.4. Differences in the Abundance Distribution of Dominant Microflora Among Different Groups of Cured Fish (At the Family Level)

**Figure 5:**
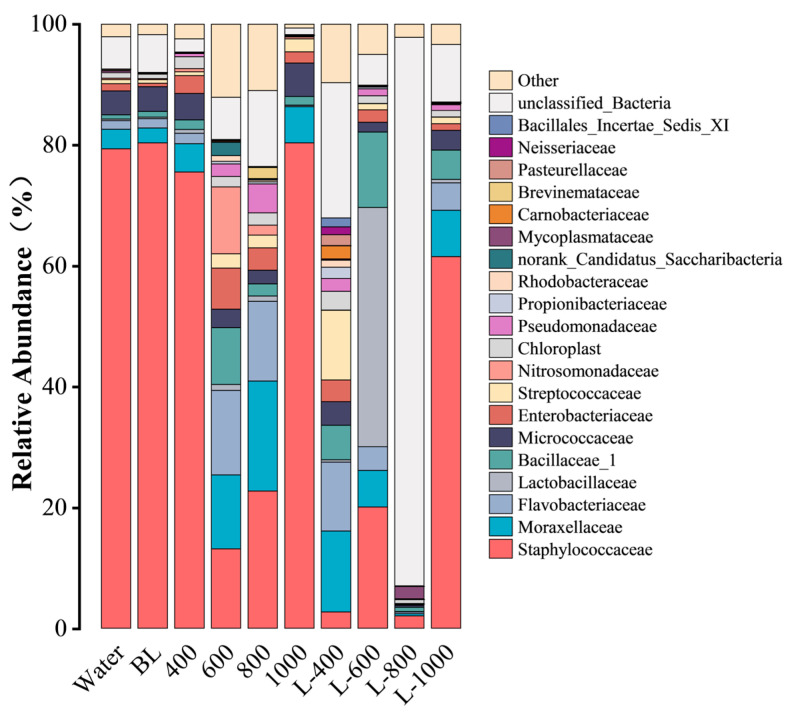
Relative abundance of dominant microflora at the family level.

Figure 5 shows the distribution of dominant microflora at the family level across the ten cured fish groups. Staphylococcaceae was the most abundant family in the Water (79.35%) and BL (80.35%) groups, followed by Moraxellaceae (3.25%, 2.47%), Micrococcaceae (3.96%, 4.12%), Enterobacteriaceae (1.20%, 0.55%), and Flavobacteriaceae (1.42%, 1.54%). This profile aligns with the representative strains isolated by traditional culture methods (e.g., *S. succinus* DZXOTU3, *S. succinus* JM40, *S. saprophyticus*, and *E. hormaechei*; Table 1).

However, the abundance of Staphylococcaceae exhibited various decreases in the other eight groups of cured fish treated with CGA solution or L-CGA. Specifically, the values were 75.54%, 13.16%, 22.73%, and 80.33% for the 400 to 1000 groups, respectively, and 2.7%, 20.08%, 2.03%, and 61.54% for the L-400 to L-1000 groups, respectively. These findings suggest that both CGA solution and L-CGA exhibit certain antimicrobial activity on the Staphylococcaceae family, and the L-CGA showed better efficacy than the corresponding CGA solutions (except 600). This trend is consistent with phylum-level observations. The only difference is that both the L-400 and L-800 groups demonstrated strong antibacterial activity against Staphylococcaceae. However, for Moraxellaceae, which was more abundant in the Water and the BL groups, the abundances in the L-400 and L-800 groups were 13.39% and 0.38%, respectively. This indicates that, compared to L-400, L-800 exhibited stronger antibacterial efficacy against Moraxellaceae.

#### 3.5.5. Differences in the Abundance Distribution of Dominant Microflora Among Different Groups of Cured Fish (At the Genus Level)

**Figure 6:**
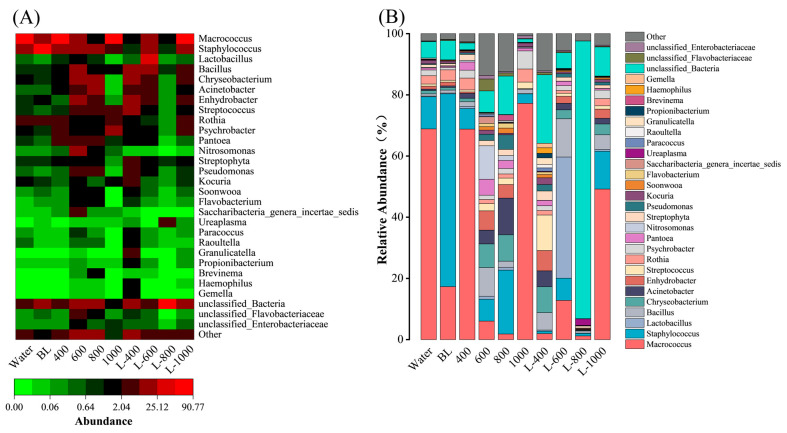
Genus-level microbial community composition (**A**) and its relative abundance with the various treatment groups (**B**) in cured fish.

The heatmap (Figure 6A) illustrates the dominant microflora at the genus level across ten cured fish samples, with the horizontal and vertical axes representing the samples and colonies, respectively, and color indicating relative abundance (red: high expression; green: low expression). The abundance of the upper half of the genera was higher than the lower half. The six predominant genera identified were *Macrococcus*, *Staphylococcus*, *Lactobacillus*, *Bacillus*, *Chryseobacterium*, and *Acinetobacter*.

In the Water group, *Macrococcus* was the dominant genus, representing 68.87% of the total microbial community. The genus *Macrococcus* is composed of eight species that are evolutionarily closely related to species of the *Staphylococcus* genus [51]. Among these, *M. elsdenii* is the most frequently detected species in fermented foods and is known for its ability to metabolize lactate into propionic acid through fermentation [52]. This metabolic process likely contributes to the reduction in pH observed during the storage of cured fish. Following treatment with L-800, the relative abundance of *Macrococcus* drastically decreased to 1.19%. This significant reduction demonstrates that L-800 can effectively inhibit the proliferation of the *Macrococcus*.

The *Staphylococcus* genus comprises a total of 55 validly published species and subspecies, such as *Staphylococcus saprophyticus* and *Staphylococcus aureus* [53]. Moreover, both *S. saprophyticus* and *S. amber* were isolated from cured fish samples using traditional cultivation techniques. Consequently, it is plausible that *S. saprophyticus* and *S. amber* are the primary *staphylococci* that proliferate and persist on cured fish during storage. This finding aligns with previous studies implying that *S. saprophyticus* is a major spoilage bacterium in food, primarily inducing spoilage through the secretion of substantial amounts of protease and lipase, which lead to protein degradation and lipid oxidation [54].

The genus *Lactobacillus*, recognized for its capability to produce lactic acid via the fermentation of carbohydrates, encompasses 261 species [55], including *Lactobacillus acidophilus*, *Lactobacillus alimentarius*, *Lactobacillus bifermentans*, and *Lactobacillus vaginalis*, etc. Diacetyl, a metabolite produced by Lactobacillus, has been shown to effectively inhibit the growth of various spoilage and pathogenic bacteria in food [56]. It has been reported to exhibit significant antibacterial activity against Gram-negative bacteria by binding to arginine in bacterial proteins, thereby interfering with arginine utilization and inhibiting bacterial growth [57].

The genus *Bacillus* can be categorized into several subgroups, including *Bacillus anthracis*, *Bacillus cereus*, *Bacillus subtilis*, *Bacillus coagulans*, and *Bacillus stearothermophilus*. Among these, Bacillus cereus is commonly found in a variety of foods, such as dairy products, fruits and vegetables, condiments, and poultry meat products [58,59]. In our study, the Bacillaceae family was identified as the predominant bacterial family during the storage of cured fish. Despite employing a two-step air-drying method (60 °C and then 8 °C) for curing fish, the spores of *Bacillus cereus* and *Bacillus stearothermophilus* retained their viability due to their significant heat resistance.

To elucidate the variations in the abundance distribution of dominant species among different groups of cured fish, a histogram was constructed and employed for data analysis (Figure 6B). In the Water group, the predominant microflora was distributed as follows: *Macrococcus* (68.87%), *Staphylococcus* (10.48%), *Rothia* (2.74%), *Psychrobacter* (1.85%), *Chryseobacterium* (1.10%), and *Kocuria* (1.10%). In contrast, within the BL group, *Staphylococcus* was the most prevalent species, comprising 63.06%, followed by *Macrococcus* (17.29%), *Rothia* (3.43%), *Enhydrobacter* (1.20%), *Psychrobacter* (0.77%), *Chryseobacterium* (0.90%), and *Kocuria* (0.66%). It is evident that although the Water and BL groups exhibited similar microbial compositions at the genus level, their dominant species differed significantly. This contrasts with the findings at the phylum and family levels, which demonstrated comparable distributions of dominant microflora abundance across these groups. The underlying reasons for these differences warrant further investigation.

The relative abundance of the genus *Megalococcus* in groups treated with 400, 600, 800, and 1000 mg/L CGA solutions was 68.80%, 6.05%, 1.83%, and 77.20%, respectively. This suggests that the 600 and 800 groups effectively inhibited the proliferation of *Megalococcus*, whereas other concentrations either had no effect or potentially enhanced *Megalococcus* growth. In contrast, all L-CGA groups (L-400 to L-1000) exhibited inhibitory effects. Furthermore, the abundance of *Macrococcus* in the L-CGA groups was generally lower than that in the corresponding CGA groups, with values of 1.94%, 12.81%, 1.19%, and 49.20%, respectively. This difference may be attributed to the protective effect of the liposomal bilayer for unstable CGA. As previously discussed, CGA is a phenolic compound characterized by an ester linkage between caffeic acid and quinic acid [29]. Its molecular structure contains a hydrolysable ester bond, oxidizable catechol group (ortho-dihydroxyphenyl), and unsaturated double bond, rendering it highly susceptible to environmental factors such as light, oxygen, heat, pH variations, and metal ions [16]. These structural characteristics make CGA prone to degradation through ester hydrolysis and oxidation. When a free CGA solution is directly applied as a dip-coating, its rapid degradation can result in reduced antioxidant and antibacterial efficacy. Conversely, encapsulating CGA within liposomes, where the phospholipid bilayer serves as a physical barrier, effectively shields the compound from these degrading factors. This encapsulation may not only enhance the stability of CGA but also extend its antimicrobial activity through the controlled release of the compound, thereby effectively inhibiting the growth of spoilage microorganisms in cured fish systems. Despite a significant portion of CGA being encapsulated within liposomes, a certain amount of unencapsulated CGA remained freely dispersed in the solution. Upon application to cured fish, the unencapsulated CGA likely exerted a crucial antimicrobial effect during the initial stages of storage, while the CGA released subsequently provided long-term protection, thereby ensuring better preservation for the L-CGA groups.

The results demonstrated that L-800 is highly suitable for application in cured fish, as it effectively suppressed the dominant microbial flora. The L-800 group exhibited the lowest total abundance of dominant bacteria after storage, with the main genera—*Macrococcus*, *Staphylococcus*, *Rothia*, *Psychrobacter*, *Chryseobacterium*, and *Kocuria*—accounting for only 1.19%, 0.84%, 0.17%, 0.069%, 0.21%, and 0.072%, respectively. This pronounced reduction highlights the strong inhibitory effect of L-800 against the typical spoilage-related bacteria in cured fish, supporting its potential as an effective antimicrobial treatment for preservation.

#### 3.5.6. The Taxonomic System Composition of Different Groups of Cured Fish

**Figure 7:**
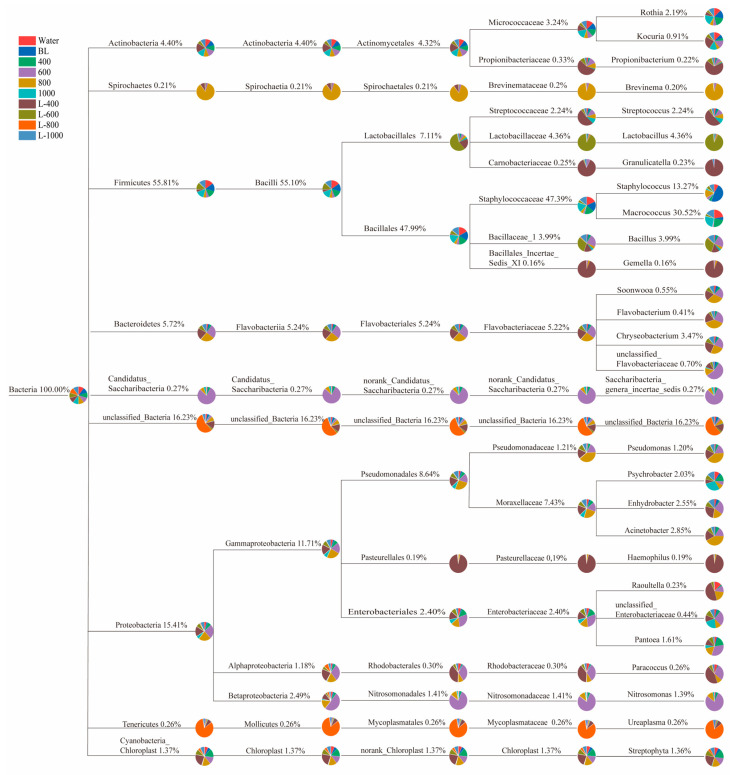
Diagram of the taxonomic system composition of microflora.

To enhance the understanding of the evolutionary relationships and variations in dominant microorganisms’ abundance within cured fish samples, a microbial taxonomy system was developed. This system facilitates comparative analysis of the microbial taxonomy across different cured fish samples. Figure 7 illustrates the composition of the taxonomic system of dominant microflora in various treated cured fish samples, with different colors representing distinct samples and the size of the sector area indicating the relative abundance of each sample. A larger sector area corresponds to a higher abundance of the microbial community within that particular branch. Clearly, Firmicutes emerged as the predominant phylum, comprising 55.81% of the microbial community, followed by Proteobacteria at 15.41%, Bacteroidetes at 5.72%, and Actinobacteria at 4.40%. Within the Firmicutes phylum, the genus *Macrococcus* exhibited the highest abundance at 30.52%, followed by *Staphylococcus* at 13.27%, *Lactobacillus* at 4.36%, *Streptococcus* at 2.24%, *Bacillus* at 3.99%, *Granulicatella* at 0.23%, and *Gemella* at 0.16%. The Proteobacteria phylum was primarily composed of *Acinetobacter* at 2.85%, *Enhydrobacter* at 2.55%, and *Psychrobacter* at 2.03%, along with *Pseudomonas* at 1.20%, *Pantoea* at 1.61%, *Nitrosomonas* at 1.39%, *Haemophilus* at 0.19%, *Raoultella* at 0.23%, and *Paracoccus* at 0.26%. Moreover, *Brochothrix* was not detected in our research. This may be attributed to variations in the growth environments for fish and the specific processing methods employed during the preparation of cured fish.

## 4. Conclusions

This study investigated the effect of CGA and L-CGA dip-coatings on the quality of low-salt cured fish during refrigerated storage. The results demonstrated that the L-CGA coating effectively maintained the physicochemical stability of the samples. Among them, the L-800 group showed the most stable pH and the most significant inhibition of lipid oxidation, with the lowest levels of POV and TBARS. Given that lipid oxidation directly causes the development of rancid odors and off-flavors, this strong inhibitory effect indicates that the L-800 coating may helps maintain the sensory quality of the low-salt cured fish during refrigerated storage, particularly its flavor and odor. Given that lipid oxidation is directly associated with the development of rancid odors and off-flavors, which are critical sensory attributes, the superior performance of the L-800 coating suggests a beneficial effect in maintaining the sensory quality (e.g., flavor and odor) of the cured fish during storage.

Regarding the microbial analysis, the predominant strains, such as *Blautia hydrotrophica*, *Enterococcus faecalis*, and *Staphylococcus succinus,* were successfully isolated and identified by traditional cultivation techniques. Subsequently, 16S rRNA HTS demonstrated that L-CGA treatment significantly suppressed the proliferation of spoilage-associated microbiota. Moreover, the relative abundances of the dominant spoilage genera, including *Macrococcus*, *Staphylococcus*, *Rothia*, and *Chryseobacterium*, were found notably diminished in the L-800 group. This effective microbial management may indirectly contribute to the preservation of sensory quality by potentially delaying spoilage-induced undesirable changes in appearance and flavor. The enhanced performance may be attributed to the protective and sustained-release properties provided by the liposomal encapsulation, which improved the stability and sustained efficiency of CGA. It needs to be noted that although L-CGA (especially L-800) represents a promising candidate as a tailored preservative for low-salt cured fish, several practical hurdles must be overcome before it can be translated into industrial applications, including the scalability of liposome production, long-term storage stability, and cost-effectiveness, etc. In addition, systematic sensory evaluation (e.g., flavor, texture, acceptability) should be carried out in future to comprehensively assess their contributions to the quality of cured fish.

## Figures and Tables

**Table 1:** Colony information.

Code	Color	Morphology	Growth Rate	Function	Identification
(A)	White	Circular, smooth surface	Relatively slow	Producing short-chain fatty acids	*Blautia hydrotrophica*
(B)	Dark yellow center, pale yellow edge	Irregular margin, crateriform elevation	Relatively fast	Undetermined	*Staphylococcus succinus* DZXOTU3
(C)	White	Circular with transparent halo	Slow	① Antimicrobial activity ② Possible spoilage organism	*Enterococcus faecalis* GI60
(D)	Yellow	Irregular margin, rough surface	Relatively fast	Liquor fermentation; Lipase production	*Staphylococcus succinic* strain JM40
(E)	White	Circular with central protrusion	Slow	Undetermined	Unidentified
(F)	Dark yellow center, pale yellow edge	Circular, smooth surface	Relatively slow	① Meat fermentation starter ② Possible spoilage organism	*Staphylococcus saprophyticus*
(G)	White	Circular, smooth surface	Extremely fast	Possible spoilage organism	*Enterobacter hormaechei*

**Table 2:** Alpha diversity analysis.

Samples	Sequences	OUTs	Shannon	Chao1	Ace	Simpson	Shannoneven	Coverage
Water	35,543.0	192.0	1.60	214.55	214.03	0.48	0.30	1.00
BL	33,620.0	175.0	1.60	196.94	189.80	0.43	0.31	1.00
400	27,926.0	190.0	1.70	205.75	206.80	0.48	0.32	1.00
600	15,186.0	247.0	4.11	253.56	251.93	0.03	0.75	1.00
800	12,358.0	259.0	3.97	267.64	266.14	0.06	0.71	1.00
1000	42,648.0	137.0	1.13	172.05	168.22	0.60	0.23	1.00
L-400	17,621.0	241.0	4.05	264.21	254.39	0.04	0.74	1.00
L-600	20,440.0	214.0	2.80	221.12	222.90	0.18	0.52	1.00
L-800	55,364.0	184.0	0.76	205.58	206.83	0.78	0.15	1.00
L-1000	221,354.0	207.0	2.48	220.14	219.40	0.26	0.46	1.00

## Data Availability

The original contributions presented in this study are included in the article. Further inquiries can be directed to the corresponding author.

## Abbreviations

The following abbreviations are used in this manuscript:

BL	Blank liposomes
Water	Distilled water
CGA	Chlorogenic acid
L-CGA	Chlorogenic acid-loaded liposome
HTS	High-throughput sequencing
POV	Peroxide value
TBARS	Thiobarbituric acid reactive substances
